# Rapid and Quantitative Detection of* Leifsonia xyli* subsp.* xyli* in Sugarcane Stalk Juice Using a Real-Time Fluorescent (TaqMan) PCR Assay

**DOI:** 10.1155/2016/2681816

**Published:** 2016-09-28

**Authors:** Hua-Ying Fu, Sheng-Ren Sun, Jin-Da Wang, Kashif Ahmad, Heng-Bo Wang, Ru-Kai Chen, San-Ji Gao

**Affiliations:** National Engineering Research Center for Sugarcane, Fujian Agriculture and Forestry University, Fuzhou, Fujian 350002, China

## Abstract

Ratoon stunting disease (RSD) of sugarcane, one of the most important diseases seriously affecting the productivity of sugarcane crops, was caused by the bacterial agent* Leifsonia xyli *subsp.* xyli* (*Lxx*). A TaqMan probe-based real-time quantitative polymerase chain reaction (qPCR) assay was established in this study for the quantification of* Lxx* detection in sugarcane stalk juice. A pair of PCR primers (Pat1-QF/Pat1-QR) and a fluorogenic probe (Pat1-QP) targeting the* Part1* gene of* Lxx* were used for the qPCR assay. The assay had a detection limit of 100 copies of plasmid DNA and 100 fg of* Lxx* genomic DNA, which was 100-fold more sensitive than the conventional PCR. Fifty (28.7%) of 174 stalk juice samples from two field trials were tested to be positive by qPCR assay, whereas, by conventional PCR, only 12.1% (21/174) were tested to be positive with a published primer pair CxxITSf#5/CxxITSr#5 and 15.5% (27/174) were tested to be positive with a newly designed primer pair Pat1-F2/Pat1-R2. The new qPCR assay can be used as an alternative to current diagnostic methods for* Lxx*, especially when dealing with certificating a large number of healthy cane seedlings and determining disease incidence accurately in commercial fields.

## 1. Introduction

Ratoon stunting disease (RSD) is one of the most widely prevalent bacterial diseases of sugarcane and has been found in most of the cane-growing areas worldwide [1]. In China, high RSD incidence (61–85%) under natural conditions was observed not only in the Nursery of Sugarcane Germplasm Resources [2, 3], but also in the major cane-growing regions [4]. RSD has become an established disease that seriously restricts the development of the cane-sugar industry in China [4]. RSD may cause a yield loss of up to 30%, while under drought conditions the estimated yield loss can be as high as 50% [1, 5]. The cumulative losses of sugarcane due to RSD have probably been greater than the losses caused by any other disease [1].

RSD of sugarcane is caused by a xylem-limited Gram-positive coryneform bacterium* Leifsonia xyli *subsp.* xyli *(*Lxx*). The causal agent was originally described as* Clavibacter xyli *subsp.* xyli *in the genus* Clavibacter* [6], based on phenotypic characteristics; but subsequently it was placed in the genus* Leifsonia* based on ribosomal RNA (rRNA) gene analysis [7]. This classification was also confirmed by Young et al. (2006) [8]. RSD was first discovered in a sugarcane cultivar Q28 in Queensland in 1944, but the causal pathogen* Lxx* was not isolated until 1980 [6]. The size of* Lxx* bacterium with straight or slightly curved rods is about 0.25–0.35 *μ*m × 1–4 *μ*m and appears to be undergoing septate division [1, 6]. The genome size is about 2.6 Mb with a GC content of about 68%, 2,044 open reading frames (ORFs), and 307 predicted pseudogenes [9]. Typically,* Lxx*-infected sugarcane plants do not show external symptoms except for the stunting and unthrifty growth, such as dwarf and fewer tillers and a thin stem [10]. RSD is mainly spread through mechanical and plant germplasm transmission [11]. Hot-water treatment (50°C for 2-3 h) is the most commonly used method to control RSD and the use of resistant clones has been a way of secondary importance [1, 12]. Apart from sugarcane, the host range of* Lxx* has been covered maize, sorghum, and numerous grasses commonly found in cane fields, but external symptoms of disease were not shown among infected plants [1].

Due to the lack of external symptoms, it is difficult for a researcher to diagnose this disease in cane fields. However, various methods have been developed to diagnose the* Lxx*. Dark-field or phase-contrast microscopes were used to detect the pathogen, but this method requires expensive devices while the sensitivity was quite low [13]. Several immunological methods were used to detect this disease, such as fluorescent antibody direct-count on filters (FADCF) [14, 15], dot blot enzyme immunoassays (DB-EIA) [16], tissue blot enzyme immunoassays (TB-EIA) [5], evaporative-binding enzyme immunoassays (EB-EIA) [17], and tissue blot DNA hybridization [18]. Molecular methods, including conventional PCR [2, 18, 19], real-time qPCR [20–23], and loop-mediated isothermal amplification (LAMP) [24, 25], have been developed for* Lxx* detection and identification. qPCR achieves high throughput, high speed, specificity, and reliability; thus it is to be the most widely applied method to diagnose and quantify plant pathogens [26, 27].

It is necessary to develop a sensitive and reliable diagnostic assay for* Lxx* in RSD control, such as monitoring transmission of the* Lxx* in cane fields and during germplasm exchanges, certificating disease-free planting material, and identifying RSD resistance in sugarcane breeding. Hence, we optimized primers' and probes' design, reaction components, and reaction conditions for* Lxx* detection using a TaqMan probe-based qPCR assay in our previous study [23]. In this study, we further analyzed the specificity and sensitivity of that assay and then used this assay to rapidly and quantitatively screen for* Lxx* in stalk juice samples from sugarcane fields.

## 2. Materials and Methods

### 2.1. Sugarcane Stalk Juice Samples

One hundred and seventy-four sugarcane juice samples were collected from 10-month-old mature stalks in sugarcane fields of two regional sugarcane cultivar trials, Fuzhou, China. For each cultivar, six cane juice samples were collected by using a hand punch from the third basal internodes of six randomly chosen plants. For each plant, one-milliliter juice sample was placed in a 1.5 mL tube. Between each collection, the hand punch was rinsed with water and then disinfected with 75% ethyl alcohol. All the samples were stored at −80°C until DNA extraction. The* Lxx*-free stalk juice was obtained from the healthy sugarcane plants growing in the greenhouse. These* Lxx*-free plants were established by routine hot-water treatment at 50°C for 2 h [12].

### 2.2. Strains of Leifsonia Species

Strain cells of* Lxx* were a kind gift of Ying Guo (Fujian Institute of Subtropical Botany, Xiamen, China). Strains cells of other three species, namely,* L. ginsengi*,* L. poae*, and* L. rubra*, were obtained from China General Microbiological Culture Collection Center (CGMCC).

### 2.3. DNA Extraction and Purification

DNA was extracted from the stalk juice samples using a cetyltrimethylammonium bromide (CTAB) method [28]. Stalk juice was centrifuged at 3,000 rpm for 5 min. 300 *μ*L supernatants were collected, mixed with 600 *μ*L CTAB buffer, and incubated at 65°C for 30 min. Then, 600 *μ*L of chloroform/isoamyl alcohol (24 : 1) was added to the mixtures, vortexed for a few seconds, and, then, centrifuged at 12,000 rpm for 10 min. Again, the upper aqueous phase was collected to a fresh microfuge tube and reextracted with chloroform/isoamyl alcohol. The aqueous phase was transferred into a microfuge tube along with 1/10 volume of 3 M sodium acetate (pH 5.2) and the same volume of isopropanol. All sample tubes were incubated at −20°C for 20 min before centrifugation at 12,000 rpm. Then DNA pellets were washed twice with 70% and 100% ethyl alcohol, respectively, and were suspended in 30 *μ*L sterile water. Bacterial genomic DNA was extracted by using Bacterial Genomic DNA Extraction Kit (Tiangen Biotech, Beijing, China) following manufacturer's instructions and eluted in 50 *μ*L sterile water. The quality and concentration of DNA were analyzed by 1% agarose gel electrophoresis and UV absorbance assay on a Synergy*™* H1 Multi-Mode Reader (BioTek, Winooski, VT, USA).

### 2.4. Primer and Probe Design

Primers and probes were designed targeting the* Pat1 *gene in strain CTCB07 (GenBank acc. number NC_006087) of* Lxx* (Table 1). Primer pair Pat1-F1/Pat1-R1 was newly designed to amplify and clone the whole 942 nucleotides (nt) of the* Pat1 *gene. The published primers CxxITSf#5/CxxITSr#5 targeted the ITS region [19] and the novel primers Pat1-F2/Pat1-R2 targeted the* Pat1 *gene. The two primer pairs were used in conventional PCR to detect* Lxx* (Table 1). Another primer pair, Pat1-QF and Pat1-QR, and a TaqMan probe (Pat1-QP) were used for real-time qPCR [23]. The TaqMan probe was labeled with 6-carboxy-fluorescein (FAM) reporter dye (excitation wavelength at 494 nm and emission wavelength at 521 nm) and 6-carboxytetramethylrhodamine (TAMRA) fluorescent quencher at 5′-end and 3′-end of the probe sequence, respectively. All primers and the TaqMan probe were synthesized by Takara Biotech (Dalian, China).

### 2.5. Conventional PCR and Real-Time qPCR

Conventional PCR amplification was carried out in a 25 *μ*L volume containing 1 *μ*L DNA, 2.5 *μ*L of 10×* EX *Taq buffer, 0.2 mM dNTPs, 0.4 *μ*M of each of the upstream and downstream primers, and 1.25 U of* EX *Taq. PCR was performed following a thermal cycling program of 94°C for 2 min; 35 cycles of 94°C for 30 s, 56°C (CxxITSf#5/CxxITSr#5) or 50°C (Pat1-F2/Pat1-R2) for 30 s, and 72°C for 60 s; and a final extension at 72°C for 10 min. For real-time qPCR, the 25 *μ*L reaction mixture contained 1 *μ*L DNA, 12.5 *μ*L TaqMan Fast Universal PCR Master Mix (Roche Applied Science, Mannheim, Germany), 2.25 *μ*L (10 pM) of each of the forward (Pat1-QF) and reverse primers (Pat1-QR), 1 *μ*L (10 pM) TaqMan probe (Pat1-QP), and 6 *μ*L sterile water. The thermal cycling conditions consisted of 50°C, 2 min, and 95°C, 10 min, and 40 cycles of 95°C, 15 s, and 60°C, 1 min. Ct values were measured in three reactions and reaction efficiency was calculated by the formula (10^−1^/slope − 1) × 100%. The Ct cut-off values were determined to be set as 35. The real-time qPCR and data analysis were performed on Applied Biosystems 7500 thermal cycler (Alameda, CA, USA).

### 2.6. Establishment of Standard Real-Time qPCR Curves

To construct the pMD19T-Pat1 plasmid, the complete 942-nt* Pat1 *gene of* Lxx* was amplified by PCR using primer pair Pat1-F1/Pat1-R1 and then subcloned into pMD19T (TaKaRa, Dalian, China). One standard curve of qPCR was generated using a 10-fold dilution series containing 10^8^ to 100 copies/*μ*L of pMD19T-Pat1 plasmid DNA. Another standard curve of qPCR was produced using a 10-fold dilution series of* Lxx* genomic DNA ranging from 100 ng to 100 fg per microliter.

### 2.7. Specificity and Sensitivity Analysis of Real-Time qPCR

The specificity of the real-time qPCR assay for the detection of* Lxx* was evaluated using the total DNA (100 ng/*μ*L) of stalk juice from* Lxx*-infected sugarcane plant,* Lxx *genomic DNA (100 pg/*μ*L), and genomic DNA (100 pg/*μ*L) of three other* Leifsonia* species, namely,* L. ginsengi*,* L. poae*, and* L. rubra*. Total DNA (100 ng/*μ*L) of* Lxx*-free stalk juice and sterile H_2_O were used as negative and blank controls, respectively. To assess the sensitivity of real-time qPCR, recombinant pMD19T-Pat1 plasmid DNA (10^8^–10 copies/*μ*L) and* Lxx* genomic DNA (from 100 ng/*μ*L to 10 fg/*μ*L) were tested by qPCR and conventional PCR in parallel. The amplified DNA fragments were analyzed by 4% or 1.5% low melting agarose gel electrophoresis, respectively.

### 2.8. Lxx Detection in Field's Stalk Juice Samples

A total of 174 field collected cane juice samples were tested for* Lxx* using real-time qPCR and conventional PCR in parallel. Ct values of less than 35 were considered* Lxx*-positive. All amplified PCR products were analyzed by 1.5% agarose gel electrophoresis and then cloned into the pMD19-T vector (TaKaRa, Dalian, China) for sequencing.

## 3. Results

### 3.1. Efficiency of Real-Time qPCR Assay

**Figure 1:**
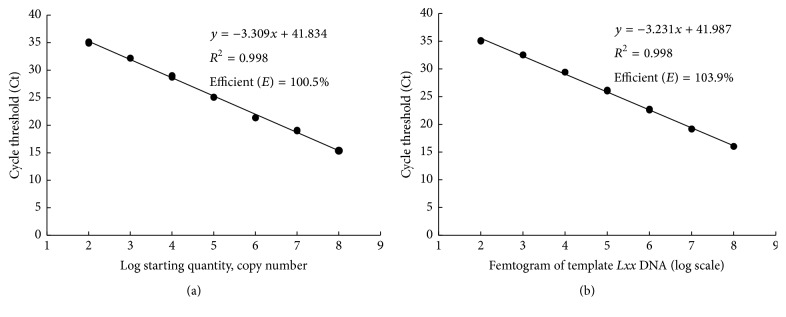
Standard curves of TaqMan probe real-time qPCR. (a) The standard curve using the templates of pMD19T-Pat1 plasmid DNA (10^8^–100 copies/*μ*L). (b) The standard curve using the templates of* Lxx* genomic DNA (100 ng/*μ*L–100 fg/*μ*L).

The standard curves of real-time qPCR on pMD19T-Pat1 were shown in Figure 1.  Figure 1(a) shows the standard curve for pMD19T-Pat1, with a slope of −3.309, efficiency (*E*) of 100.5%, and *R* ^2^ = 0.998.  Figure 1(b) shows the standard curve for of* Lxx* genome DNA, with a slope of −3.231, efficiency (*E*) of 103.9%, and *R* ^2^ = 0.998. Both *E* and slope reached their specifications (*E* = 95–105%, slope close to −3.3) for real-time qPCR. According to the cut-off values of Ct = 35, the detection limit of the real-time qPCR assay was 100 copies for pMD19T-Pat1 (Figure 1(a)) and 100 fg for* Lxx* genomic DNA (Figure 1(b)).

### 3.2. Specificity and Sensitivity of Real-Time qPCR

**Figure 2:**
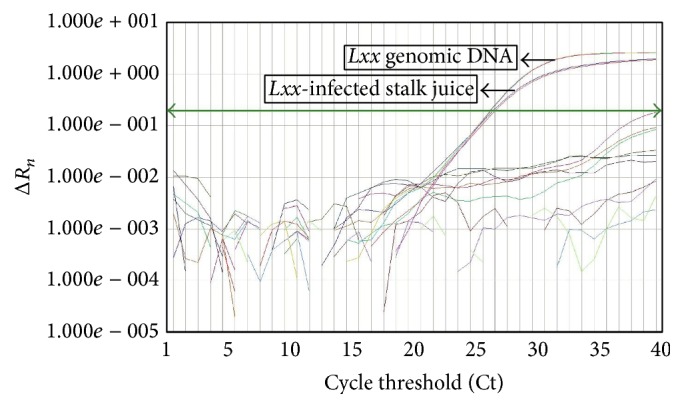
Specificity of real-time qPCR with TaqMan probe using individual DNA templates, total DNA (100 ng) of* Lxx-* (*Leifsonia xyli *subsp.* xyli-*) infected stalk juice, bacterial genomic DNA (100 pg) of* Lxx*,* L. ginsengi*,* L. poae*, and* L. rubra*, and total DNA (100 ng) of* Lxx*-free stalk juice (negative control) and sterile H_2_O (blank control). Three technical replicates were used in each DNA concentration.

**Figure 3:**
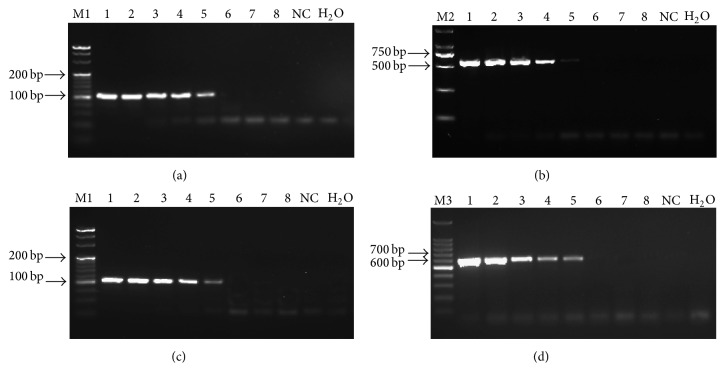
Sensitivity tests based on 4% ((a) and (c)) or 1.5% ((b) and (d)) agarose gel electrophoresis of the amplicons from plasmid DNA (pMD19T-Pat1) and genome DNA of* Leifsonia xyli *subsp.* xyli *(*Lxx*) using two set primers of qPCR (Pat1-QF/Pat1-QR) ((a) and (c)) and conventional PCR (Pat1-F2/Pat1-R2) ((b) and (d)). The same set of diluted solutions (10^8^–10 copies/*μ*L) of plasmid DNA (pMD19T-Pat1) were carried out in (a) and (b), and the same set of templates (100 ng/*μ*L–10 fg/*μ*L) of genome DNA of* Lxx* were used in (c) and (d). M1: 20 bp DNA ladder; M2: DL 2000 DNA marker; M3: 100 bp DNA ladder; NC: total DNA (100 ng/*μ*L) extracted from* Lxx*-free sugarcane stalk juice.

The real-time qPCR assay did not produce any amplification products from the three other* Leifsonia *species, total DNA from a* Lxx*-free plant, and sterile H_2_O but produced two typical amplification curves from the total DNA of stalk juice DNA samples of* Lxx*-infected sugarcane plants and from* Lxx *genomic DNA (Figure 2), indicating that the novel primers and probe were specific to* Lxx* detection. For the sensitivity analysis of real-time qPCR, two amplification fragments of 109 bp and 596 bp in size were observed in conventional PCR, by 4% and 1.5% agarose gel electrophoresis, respectively (Figure 3). The detection limit was 1 × 10^4^ copies of pMD19T-Pat1 plasmid DNA for both primer pairs Pat1-QF/Pat1-QR (Figure 3(a)) and Pat1-F2/Pat1-R2 (Figure 3(b)). Besides, the detection limit on* Lxx* genomic DNA was 10 pg by either primer pair, that is, Pat1-QF/Pat1-QR (Figure 3(c)) or Pat1-F2/Pat1-R2 (Figure 3(d)). Comparing with the detection limit of 100 copies for pMD19T-Pat1 plasmid DNA (Figure 1(a)) and 100 fg for* Lxx* genomic DNA (Figure 1(b)) by real-time qPCR assay, we proposed that the real-time qPCR was 100-fold more sensitive than the conventional PCR.

### 3.3. Detection of Lxx in Field Stalk Juice Samples

Of 174 cane juice samples from the field, 50 (28.7%) were tested to be positive for* Lxx* infection by real-time qPCR method, whereas, by conventional PCR, 21 (12.1%) were tested to be positive using primers pair CxxITSf#5/CxxITSr#5, while 27 (15.5%) were tested to be positive using primer pair Pat1-F2/Pat1-R2, respectively (Table 2). The qPCR results revealed that the* Lxx* titer varied from 1.15 × 10^2^ to 2.11 × 10^4^ copies per microliter of total juice DNA template. More than 50% incidence of* Lxx*-positive juice samples was found in four varieties (ROC16, MT06-1045, YG45, and YG47) irrespective of using qPCR or conventional PCR. However, five sugarcane varieties, FN07-3206, LC07-500, YR07-1433, YZ07-2384, and HZ22, were found to be* Lxx*-free under natural conditions by qPCR assay. To exclude the possibility of nonspecific amplification, all qPCR and conventional PCR products were cloned and sequenced. All the sequences were found to be 100% identical to corresponding* Lxx* sequences using the BLASTN tool (http://blast.ncbi.nlm.nih.gov/).

## 4. Discussion

Sugarcane is an economically important crop for contributing to a majority of the sugar production in the world. It is a critical method using healthy seedling for RSD control for avoiding significant crop yield loss. To prevent RSD from spread and dissemination of the pathogen through infected plant cuttings or germplasm, a TaqMan probe-based qPCR assay was used in this study for the quantification of* Lxx* detection in sugarcane stalk juice samples. Unlike the conventional PCR, qPCR, a high-throughput technique, allows pathogen quantification and monitoring of the reaction while in progress and overcomes the risk of cross contamination through modified primers and different labels of primers in combination with probes. Different chemistries for qPCR are commercially available including SYBR Green, TaqMan, Scorpion, and Molecular Beacons, but TaqMan is the most used real-time system, since it can discriminate sequences that differ by only one nucleotide [27]. Hence, a TaqMan-based qPCR assay for* Lxx* in sugarcane stalk juice has been optimized in our previous study [23], and, in this study, we used this established qPCR assay to rapidly and accurately screen for the presence of* Lxx* in stalk juice samples collected from the sugarcane fields.

The genus* Leifsonia *belongs to the family Microbacteriaceae and comprises 11 species and two subspecies (*Lxx* and* Leifsonia xyli *subsp.* cynodontis*) [29]. The similarities of the 16S rRNA gene sequences among* Leifsonia *species ranged from 94 to 99.8%, and the inferred phylogenies showed that the genus comprises a rather heterogeneous group [29]. However, the ITS and 16S rDNA sequences among* Lxx* isolates were found to be extremely identical. Young et al. (2006) reported that no variation was detected among 105 different* Lxx* isolates from nine countries using DNA fingerprinting [8]. Furthermore, no sequence variation was observed within the ITS region of the ribosomal RNA genes from 54 isolates using both single-stranded conformational polymorphism (SSCP) and direct sequencing. Similarly, 99.5–100% or 100% identities were shared among the Chinese* Lxx* isolates [2–4]. In this study, the* Pat1 *gene was chosen for designing the primers and TaqMan probe because its sequence had no significant similarity (<90%) to any other available sequences, including the subspecies of* Leifsonia xyli *subsp.* cynodontis* [30] in the GenBank library.* Leifsonia xyli *carries a gene* Pat-1 *that is similar to* Clavibacter michiganensis *subsp.* michiganensis*, which plays an important role in causing plant wilting [9, 31].

One of the crucial characteristics of TaqMan-based qPCR assay is its high specificity. Hence, to verify the specificity of the primers and probe used in this study, three other* Leifsonia *species, namely,* L. ginsengi*,* L. poae, *and* L. rubra*, were included. The results showed that no fluorescent signal had ever been detected (Figure 2). On the other hand, when the sequences of qPCR primers and probe were aligned with the putative* Pat1 *gene sequence of* L. xyli *subsp.* cynodontis*, poor base-pair matching was observed. Hence, the newly developed qPCR assay would not work on* Leifsonia xyli *subsp.* cynodontis *detection. We proposed that the TaqMan probe Pat1-QP and the primers Pat1-QF and Pat1-QR are highly specific for* Lxx* detection.

Another characteristic of the real-time qPCR is its sensitivity. The newly developed qPCR assay for* Lxx* was 100-fold more sensitive than the conventional PCR on either pMD19T-Pat1 plasmid DNA or bacterial genomic DNA. Pan et al. (1998) reported that the minimum detection limit of their conventional PCR protocol was 100 pg* Lxx* genomic DNA using primer pair Cxx1 and Cxx2 that targeted the 16S–23S rDNA ITS [18]. The primer pair CxxITSf#5 and CxxITSr#5 designed by Fegan et al. (1998) could detect as few* Lxx* cells as 1.1 × 10^4^/mL using conventional PCR assay [19]. Subsequently, Grisham et al. (2007) established SYBR real-time qPCR method to effectively detect* Lxx* in infected leaves throughout the growing season [20]. Another SYBR-based qPCR was developed to quantify the effects of heat treatment of sugarcane cuttings on* Lxx* [21]. Recently, Pelosi et al. (2013) developed a TaqMan-based qPCR assay for* Lxx* detection in the stalk or central veins of leaves and also compared between conventional PCR and nested-PCR assays [22]. Their findings also showed that qPCR was more sensitive than conventional PCR but nearly as sensitive as the nested-PCR. However, qPCR had more advantages than nested-PCR when processing a large number of samples, because it did not require postprocessing steps for visualization of results. In addition, the level of bacterial infection could be quantified [22]. More recently, two LAMP assays were developed to diagnose as little as 3 pg* Lxx* genome DNA [24, 25]. Comparing with all the studies cited above, the TaqMan-based qPCR assay developed in this study was more sensitive than conventional PCR or had similar sensitivity with LAMP assays involving* Lxx* genomic DNA. The qPCR assay developed in this study had a detection limit of 100 copies of* Lxx* genomic DNA, which was 10-fold less sensitive than the qPCR assay developed in the previous report [22]. This difference could be due to the primers and probes used in the two cases.

When the TaqMan-based qPCR assay was used to screen the incidence of* Lxx* infection in field juice samples, more positive samples were observed by qPCR compared to conventional PCR. We have excluded false-positive results through sequencing of all PCR products. It is noteworthy that all the PCR products from positive samples shared a 100% identity with the corresponding genomic regions of* Lxx*. In addition, five sugarcane cultivars (FN07-3206, LC07-500, YR07-1433, YZ07-2384, and HZ22) in two field trials were not infected by* Lxx* under natural conditions, whereas these cultivars need to be further tested for RSD resistance by artificial inoculation. We observed that the overall incidence of RSD in tested sugarcane cultivars was lower in this study compared to those of previous studies [2–4]. One reason would be different sugarcane clones being tested in different studies. Another reason may be different* Lxx* inoculum pressure under natural conditions, because Yunnan province is the second top commercial sugarcane producer in China. In contrast, Fujian province, particularly Fuzhou city, has had little commercial sugarcane fields since the early 1990s.

## 5. Conclusion

In this study, a TaqMan qPCR assay was used to diagnose the presence of* Lxx* in sugarcane stalk juice samples. This assay was more convenient when working with DNA samples extracted directly from the stalk juice by omitting the step of leaf tissue grinding and reducing the amount of PCR inhibitors. This assay provides important technical support for RSD epidemic surveillance and ecological prevention and control through certifying healthy seed cane program and determining accurate RSD incidence in the commercial fields.

## Figures and Tables

**Table 1:** Primers and probe information for *Leifsonia xyli *subsp. *xyli* (*Lxx*).

Primer/probe	Sense	Sequence (5′-3′)^a^	Gene target^b^	Amplicon size (bp)	Reference
Pat1-F1	Forward	TTGTTTAGTTTTCGTTGGCG	*Pat1*	942	In this study
Pat1-R1	Reverse	CTATGCTGGAGCCACAG			
CxxITSf#5	Forward	TCAACGCAGAGATTGTCCA	ITS	305	Fegan et al., 1998 [19]
CxxITSr#5	Reverse	GTACGGGCGGTACCTTTTC			
Pat1-F2	Forward	GGAATACTCGCTATGTGTTG	*Pat1*	597	In this study
Pat1-R2	Reverse	CCAATACTATGCCGTAGAAAG			
Pat1-QF	Forward	GGTTCCATTGCTTACCGATT	*Pat1*	106	Wang et al., 2015 [23]
Pat1-QR	Reverse	CAAGTTTCGACAGGAACAGC			
Pat1-QP	Probe	FAM-CCACGGCTACGTCAATTCGGG-TAMRA			

^a^FAM, 6-carboxy-fluorescein reporter dye; TAMRA, 6-carboxytetramethylrhodamine fluorescent quencher.

^b^Primers and probe targeted at *Pat1* gene of *Leifsonia xyli* subsp. *xyli* (strain CTCB07, GenBank acc. number NC_006087) were designed by authors of this study and Wang et al. [23]. CxxITSf#5/CxxITSr#5 targeted at 16S-23S rRNA internal transcribed spacer (ITS) regions reported by Fegan et al. [19].

**Table 2:** Detection of *Leifsonia xyli* subsp. *xyli* (*Lxx*) in stalk juice samples from sugarcane fields.

Trial	Variety	Conventional PCR		qPCR	
		CxxITSf#5/CxxITSr#5	Pat1-F2/Pat1-R2	Pat1-QF/Pat1-QR	Concentration of DNA (copies/*µ*L)
Trial #10	FN07-2020	1/6	1/6	1/6	7.65 × 10^2^
	FN07-3206	0/6	0/6	0/6	na
	FN40	1/6	1/6	1/6	2.19 × 10^2^
	GZ07-538	0/6	0/6	1/6	1.90 × 10^2^
	LC07-500	0/6	0/6	0/6	na
	LC07-536	0/6	0/6	1/6	1.42 × 10^2^
	MT02-205	0/6	0/6	2/6	1.20 × 10^2^–1.85 × 10^2^
	ROC16	4/6	3/6	5/6	2.40 × 10^2^–4.02 × 10^2^
	ROC22	0/6	0/6	0/6	na
	YG43	0/6	0/6	1/6	1.94 × 10^2^
	YG46	1/6	3/6	4/6	2.92 × 10^2^–1.11 × 10^3^
	YR07-1433	0/6	0/6	0/6	na
	YZ07-2384	0/6	0/6	0/6	na
	YZ08-2060	0/6	0/6	1/6	1.62 × 10^2^

Trial #11	DHZ07-36	0/6	0/6	1/6	1.16 × 10^2^
	FN09-0906	0/6	0/6	1/6	2.13 × 10^3^
	FN09-7111	0/6	2/6	3/6	2.98 × 10^2^–2.11× 10^4^
	GT06-2081	0/6	0/6	1/6	4.04 × 10^2^
	GT07-25	0/6	1/6	2/6	4.11 × 10^2^–1.75 × 10^4^
	GT08-1180	0/6	0/6	1/6	1.59 × 10^2^
	HZ22	0/6	0/6	0/6	na
	LC07-150	0/6	1/6	2/6	1.23 × 10^3^–1.29 × 10^3^
	MT06-1045	3/6	4/6	4/6	1.09 × 10^2^–5.47 × 10^3^
	ROC22	0/6	0/6	1/6	5.61 × 10^2^
	YG45	4/6	4/6	5/6	1.73 × 10^2^–5.54 × 10^3^
	YG47	6/6	6/6	6/6	1.28 × 10^2^–1.16 × 10^4^
	YR06-241	0/6	0/6	3/6	1.15 × 10^2^–1.98 × 10^3^
	YZ08-1095	0/6	0/6	1/6	2.94 × 10^3^
	YZ08-1145	1/6	1/6	2/6	4.21 × 10^2^–3.75 × 10^3^

na, data not available.
